# 3D and 4D Printing of PETG–ABS–Fe_3_O_4_ Nanocomposites with Supreme Remotely Driven Magneto-Thermal Shape-Memory Performance

**DOI:** 10.3390/polym16101398

**Published:** 2024-05-14

**Authors:** Kiandokht Mirasadi, Davood Rahmatabadi, Ismaeil Ghasemi, Mohammad Khodaei, Majid Baniassadi, Mahdi Bodaghi, Mostafa Baghani

**Affiliations:** 1 School of Mechanical Engineering, College of Engineering, University of Tehran, Tehran 14155-6619, Iran; kmirasadi322@gmail.com (K.M.); d.rahmatabadi@ut.ac.ir (D.R.); m.baniassadi@ut.ac.ir (M.B.); 2 Faculty of Processing, Iran Polymer and Petrochemical Institute, Tehran 14965-115, Iran; 3 Materials Engineering Group, Golpayegan College of Engineering, Isfahan University of Technology, Golpayegan 87717-67498, Iran; m.khodaei@iut.ac.ir; 4 Department of Engineering, School of Science and Technology, Nottingham Trent University, Nottingham NG11 8NS, UK

**Keywords:** nanocomposite, shape-memory polymers, magneto-thermomechanical properties, 4D printing, FDM

## Abstract

This study introduces novel PETG–ABS–Fe_3_O_4_ nanocomposites that offer impressive 3D- and 4D-printing capabilities. These nanocomposites can be remotely stimulated through the application of a temperature-induced magnetic field. A direct granule-based FDM printer equipped with a pneumatic system to control the output melt flow is utilized to print the composites. This addresses challenges associated with using a high weight percentage of nanoparticles and the lack of control over geometry when producing precise and continuous filaments. SEM results showed that the interface of the matrix was smooth and uniform, and the increase in nanoparticles weakened the interface of the printed layers. The ultimate tensile strength (UTS) increased from 25.98 MPa for the pure PETG–ABS sample to 26.3 MPa and 27.05 MPa for the 10% and 15% Fe_3_O_4_ nanocomposites, respectively. This increase in tensile strength was accompanied by a decrease in elongation from 15.15% to 13.94% and 12.78%. The results of the shape-memory performance reveal that adding iron oxide not only enables indirect and remote recovery but also improves the shape-memory effect. Improving heat transfer and strengthening the elastic component can increase the rate and amount of shape recovery. Nanocomposites containing 20% iron oxide demonstrate superior shape-memory performance when subjected to direct heat stimulation and a magnetic field, despite exhibiting low print quality and poor tensile strength. Smart nanocomposites with magnetic remote-control capabilities provide opportunities for 4D printing in diverse industries, particularly in medicine, where rapid speed and remote control are essential for minimally invasive procedures.

## 1. Introduction

Additive manufacturing (AM) or three-dimensional (3D) printing is a rapidly growing technology in manufacturing and material production [1]. This groundbreaking technology was initially introduced by Charles Hull in 1986 through a process called stereolithography. Subsequently, it underwent significant advancement through extensive research and development [2]. AM offers several advantages over traditional manufacturing techniques, such as the elimination of design limitations and the ability to use design templates. Fused deposition modeling (FDM) is a popular 3D-printing technology due to its fast production, ease of operation, and cost-effectiveness [3]. By incorporating shape-memory polymers (SMPs) into the FDM printing process, new research opportunities can arise in the field of production, leading to advancements in four-dimensional (4D) printing technology [4,5]. These polymers allow 3D-printed products to respond to external stimuli [6,7].

SMPs are a unique type of smart material that can return to its original shape when exposed to a specific stimulus such as heat [8], an electric field [9], a magnetic field [10,11], or light [12,13]. In recent years, polymer composites have gained significant attention for their ability to enhance different properties of the polymer matrix, such as strength, modulus, physical properties, and heat resistance [14]. SMP composites have transformed material science with their distinctive features, allowing for their use in a variety of fields. Among those composites, those that respond to magnetic stimuli have proven to be exceptionally advantageous [15]. The ability to remotely control their behavior has opened up new horizons, allowing for innovations that were previously impossible [16]. In situations where direct thermal stimulation of the SMP is not feasible, the application of an alternating magnetic field can activate the shape-memory effect (SME) of these materials. When an SMP composite is responsive to magnetic stimuli, the ferromagnetic particles change location and align with the direction of the magnetic field lines [17]. One of the most compelling features of magnetically triggered SMPs is their ability to generate an internal rise in temperature within the polymers, rather than just heating the surface. This unique characteristic leads to rapid and significant temperature increases. Interest in magnetically activated shape-memory materials (SMMs) has surged, particularly in the biomedical field. This is attributed to their capability of being remotely controlled without the requirement of heat, making them safer and less invasive when used in the body [15].

**Figure 1:**
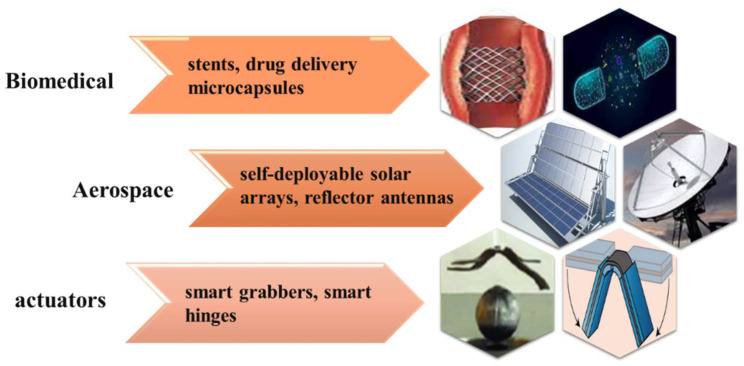
Remote control applications of magnetic SMPs.

Embedded magnetic particles, such as iron oxide (Fe_3_O_4_), can generate inductive heating when exposed to alternating magnetic fields. This phenomenon has been explored in various fields, including biomedicine, aerospace, and actuators. Figure 1 illustrates some magnetically responsive SMPs effectively utilized in remote control applications. The use of magnetic remote control holds promise in biomedical applications, since most living systems are not sensitive to magnetic fields, posing a relatively small threat to human beings. This opens new possibilities for telemedicine and diagnostic equipment. These nanocomposites exhibit superparamagnetic properties. Moreover, an increase in temperature, known as hyperthermia, has been recognized as a vital clinical tool for cancer cell therapy [18]. This underscores the importance of conducting further research in this area to address this critical aspect. Additionally, the SME allows for potential applications of magnetic nanocomposites in intravenous and localized administration through minimally invasive approaches [19,20].

The groundbreaking work of Mohr et al. [21] involved incorporating Fe_2_O_3_ nanoparticles into a thermoplastic SMP composite. They found that these particles were uniformly distributed throughout the SMP matrix. By subjecting the composite to inductive heating with an alternating magnetic field at a frequency of 258 kHz, they observed a gradual increase in temperature, which activated the shape-memory effect (SME). The maximum temperature achieved depended on the sample’s geometry, with some samples reaching temperatures up to 70 °C. These findings demonstrated the potential of using magnetism for indirect thermal induction.

Fe_3_O_4_ nanoparticles are extensively researched due to their magnetic properties, which enable them to respond to magnetic fields [22]. Zhang et al. [23] conducted a study in which they 3D-printed various structures using a biocompatible PLA–Fe_3_O_4_ nanocomposite. They investigated the shape-memory behaviors induced by alternating magnetic fields (at 27.5 kHz) on the 4D-printed structures. This research highlighted the potential of these structures in biological and medical applications, particularly in bone tissue repair. Zhao et al. [24] introduced PLA–Fe_3_O_4_ SMP composites as scaffolds for bone tissue. Their research showed that these scaffolds can be effectively stimulated by magnetic fields, resulting in significantly improved cell attachment. Huang et al. [25] developed and processed a multi-stimulus-responsive intelligent PLA–ENR–Fe_3_O_4_ composite through dynamic vulcanization. Their work produced composite materials with excellent shape-memory properties (Rr 97.72%) when the Fe_3_O_4_ content was 30 phr. Most research in the field of 4D printing using the FDM method has primarily focused on PLA due to limitations in available primary materials. This issue is related to the excellent printing ability of PLA, as mentioned earlier. In order to achieve magnetic stimulation, approximately 10–30% by weight of nanoparticles must be added to the polymer matrix, which further exacerbates the printing limitations. Additionally, it is important to note that the interfaces between metal magnetic nanoparticles and the polymer matrix are very weak.

Polyethylene terephthalate glycol (PETG) is a widely used 3D-printing filament known for its excellent printability and strong layer adhesion [26]. Its remarkable properties include high abrasion resistance and transparency, making it ideal for various applications in the biomedical field. These applications include the production of bone tissue-engineering scaffolds and tooth aligners, highlighting the indispensability of PETG in this innovative and crucial industry [25]. Soleyman et al. [27] recently conducted a study on the potential of PETG for 4D printing. Their research revealed that PETG is a promising thermoplastic with shape-memory capabilities, and it exhibits excellent printability using FDM technology. However, PETG faces a challenge due to the tendency of its molecular chains to slide when exposed to heat up to its glass transition temperature (T_g_). This limits the material’s ability to retain strain effectively, leading to a high relaxation rate after the programming stage [28]. Mirasadi et al. addressed the challenge of enhancing the structural integrity of 4D-printed objects by using a mixture of PETG and acrylonitrile butadiene styrene (ABS) as raw materials. According to their research, blending PETG and ABS creates a PETG–ABS composite material with two distinct transition temperatures, ranging from 80 to 110 degrees Celsius, which are specific to each component. ABS, with a higher T_g_ compared to PETG, enhances the formation of stronger interfaces and prevents the slipping of PETG chains, ultimately enhancing the mechanical stability of the 4D-printed objects [29].

For the first time, this paper presents the design, processing 3D printing, and 4D printing of a novel PETG–ABS–Fe_3_O_4_ nanocomposite. Three different weight percentages of iron oxide nanoparticles were incorporated to enhance the shape-memory performance and enable indirect stimulation through a magnetic field. In order to tackle the challenges related to filament production and printing of nanocomposites with a high filler weight percentage, a pneumatic system was utilized to regulate the melt flow. The mechanical properties, thermal analysis, morphology, printability, and direct and indirect thermoresponsive performance of the nanocomposites were evaluated through tensile testing, dynamic mechanical thermal analysis (DMTA), scanning electron microscopy (SEM), and shape recovery cycle in water and a magnetic coil.

## 2. Materials and Methods

### 2.1. Materials

In this study, PETG filament with a diameter of 1.75 mm was obtained from eSUN Company in Shenzhen, China, while ABS granules were sourced from Golpayegan Petrochemical Company in Iran. Spherical iron oxide nanoparticles with particle size of 20–40 nm and chemical composition of Fe_3_O_4_ were purchased from Fine Nano in the United States. The PETG filament was crushed in a plastic mill. Afterward, the raw materials were subjected to a dehumidification process in an oven at 60 °C for 6 h.

### 2.2. Processing of PETG–ABS–Fe_3_O_4_ Nanocomposites

**Figure 2:**
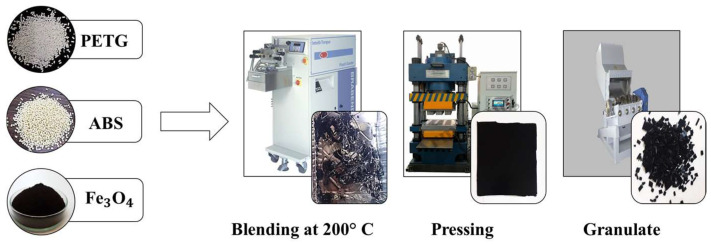
Preparation steps of PETG–ABS–Fe_3_O_4_ nanocomposites.

In this study, three types of PETG–ABS–Fe_3_O_4_ nanocomposites were produced, each containing 10%, 15%, and 20% by weight of Fe_3_O_4_ nanoparticles. These composites were based on a polymer matrix consisting of 70% by weight PETG and 30% by weight ABS. As shown in Figure 2, the raw materials were melted and mixed using a Brabender internal mixer from Germany. The process involved pouring PETG granules into the mixer at 200 °C for two minutes until they were completely melted, followed by addition of ABS granules and iron oxide nanoparticles. The mixture was left in the device for 10 min to ensure a uniform distribution of the nanoparticles in the polymer matrix. A higher shear rate was used to achieve a more uniform distribution of nanoparticles by applying a speed of 100 rpm. The output materials were lumpy polymer blends. Sheets were prepared from the mixed materials using a hot-press machine at a temperature of 200 °C for 10 min, then they were pressed for another 10 min under a pressure of 60 kPa in a cold-press machine. Finally, the produced sheets were turned into granules to prepare for printing.

### 2.3. 3D Printing

**Figure 3:**
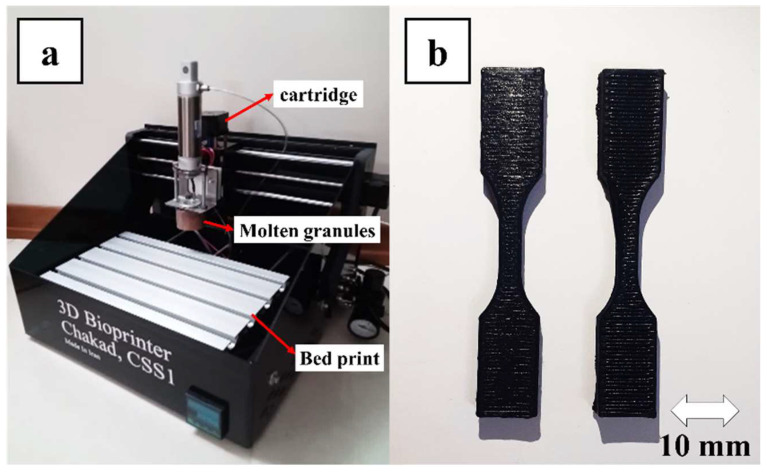
(**a**) A direct pellet-based FDM printer, (**b**) 10%Fe_3_O_4_ printed samples.

The PETG–ABS–Fe_3_O_4_ nanocomposites were printed using the FDM method with a new-generation printer. Unlike traditional printers that require filament extraction for printing, this printer operates by feeding raw-material granules directly into the thermal chamber. According to Figure 3a, once inside the cartridge, the granules are melted and reach suitable rheology in a semi-melted form before being guided into the nozzle using air pressure for printing. In addition to eliminating the filamentation step, this system also helps us achieve higher printing capabilities [29]. Printing nanocomposites with a high weight percentage of filler presents the main challenge of nozzle clogging and high porosity. Figure 3b displays the printed samples. The basic parameters of FDM printing for these nanocomposites are outlined in Table 1.

### 2.4. Characterizations

#### 2.4.1. DMTA

The dynamic viscoelastic properties and thermal behavior of PETG–ABS–Fe_3_O_4_ nanocomposites were investigated in this study. Different weight percentages of Fe_3_O_4_ nanoparticles were used and the experiments were conducted using a Mettler Toledo dynamic thermomechanical device from Switzerland. The measurements were taken over a temperature range of −100 °C to 100 °C at a constant frequency of 1 Hz, following the ASTM D4065-01 standard [30] for three-point bending load on a rectangular sample with dimensions of 1 × 30 × 5 mm.

#### 2.4.2. Microstructure Evaluation

The matrix morphology, interface quality of the 3D-printed layers of nanocomposites, and the distribution of Fe_3_O_4_ nanoparticles in the matrix were studied using SEM imaging. Prior to imaging, the samples were fractured in liquid nitrogen and then coated with gold. The images were captured using the Vegall model SEM manufactured by Tescan. Also, in order to evaluate the morphology of the matrix, the etching method was used before SEM imaging. In this method, a cross section of the PETG–ABS blend and PETG–ABS–Fe_3_O_4_ nanocomposite containing 20% by weight of iron oxide was placed in acetone to dissolve the ABS phase.

#### 2.4.3. Mechanical Properties

The mechanical properties of PETG–ABS–Fe_3_O_4_ nanocomposites were assessed at room temperature and under tensile loading, with a displacement rate of 3 mm/min. The evaluations were conducted using a Santam machine with a 5-ton capacity and a 100 kg load cell following the ASTM D638 type V standard [31]. To ensure accurate results, each sample was tested three times.

#### 2.4.4. Shape-Memory Effect (SME)

**Figure 4:**
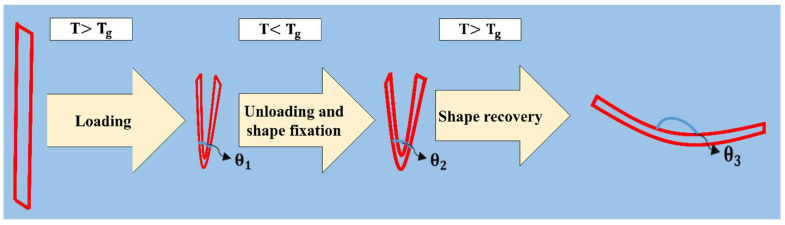
Schematic of shape-memory cycle in bending loading mode.

The PETG–ABS–Fe_3_O_4_ nanocomposites were assessed for their SME in bending loading mode, which involved programming and recovery stages. The programming process included heating, loading, cooling, and unloading of the printed samples. After loading and unloading, the bending angles (θ_1_ and θ_2_) were determined as shown in Figure 4. The shape fixity (R_f_) which indicates the ratio of fixed strain to the applied strain, can be determined using the following equation:

$$R_{f}=\frac{180-\theta_{1}-\theta_{2}}{180-\theta_{1}}\times 100$$

To restore the original shape, the deformed samples were reheated to a temperature above T_g_. Once the sample temperature reached the thermal range between the glass transition and the rubber region, the shape recovery process began. The values of the bending angle after recovery (θ_3_) are shown in Figure 4. The shape recovery parameter is also calculated using the following formula:

$$R_{r}=\frac{180-\theta_{1}-\theta_{3}}{180-\theta_{1}-\theta_{2}}\times 100$$

The test parts were beam-shaped with dimensions of 4 × 1 cm and a thickness of 1 mm. The samples were heated in water at 100 °C for 120 s, then subjected to bending loading. After that, the samples were stabilized in water at 0 °C and the load was removed. The recovery process involved both direct and indirect application of heat. For direct application, water at a temperature of 100 °C was used. Indirect thermal shape recovery was assessed by a high frequency induction heater. This device has two main parts: inverter and coil. The coil has 7.5 spirals with a diameter of 50 mm and wire diameter of 5mm. An inverter with a nominal power of 1800 W (input 15–60 V and 5–60 A) and an operating frequency of 100 kHz was used. To ensure accuracy, shape recovery tests were repeated three times for all samples.

## 3. Results and Discussion

### 3.1. Thermal Analysis

**Figure 5:**
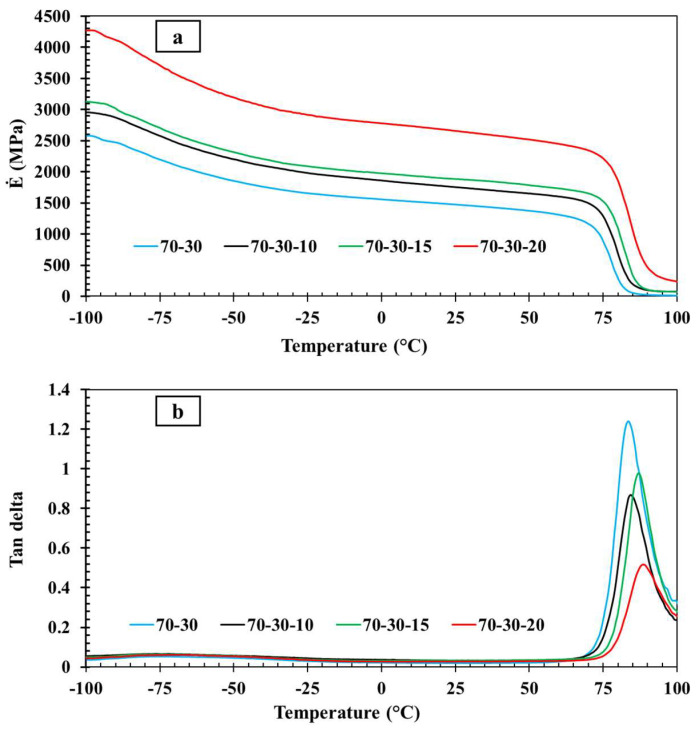
Results of thermal analysis: (**a**) Storage modulus diagrams and (**b**) tan delta of PETG–ABS–Fe_3_O_4_.

To obtain a more accurate evaluation of the shape-memory behavior, it is crucial to analyze the thermal analysis results to determine the T_g_ and different thermal regions of the material. Additionally, analyzing the DMTA results can help assess the impact of Fe_3_O_4_ nanoparticles on the viscoelastic properties of composites. Figure 5 illustrates the storage modulus and Tan delta diagrams of PETG–ABS–Fe_3_O_4_ nanocomposites with varying weight percentages of –Fe_3_O_4_ across a temperature range of −100 °C to 100 °C. Due to its immiscibility, PETG–ABS has two tan delta peaks at temperatures of 84 °C and 116 °C, which are related to the transition temperature of its constituents—PETG and ABS, respectively. According to Figure 5b, the tan peak is observed in the range of 84–87 °C, which is due to the larger volume of PETG content in all three composites and the measured temperature range. In fact, all three composites have an immiscible morphology with two tan delta peaks related to the contents, and the addition of Fe_3_O_4_ did not affect the compatibility of the blend. Figure 5a shows that an increase in the weight percentage of Fe_3_O_4_ in the PETG–ABS blend resulted in higher storage modulus values in the glassy region. Specifically, the storage modulus of the sample with 20% Fe_3_O_4_ by weight was approximately 4250 MPa, indicating a 1750 MPa increase compared to the pure matrix without nanoparticles. This represents a 41% increase in storage modulus, which can be attributed to enhanced interactions between the nanoparticles and the polymer matrix. Essentially, the material’s strength at low temperatures (below 50 °C) increased with higher Fe_3_O_4_ content. According to the results in Figure 5b, the peak Tan delta value for the PETG–ABS and PETG–ABS–Fe_3_O_4_ samples is around 83 °C to 87 °C corresponding to the T_g_. Increasing the percentage of Fe_3_O_4_ in the matrix also increases the T_g_ of the composites due to the strong interaction between Fe_3_O_4_ and the PETG–ABS matrix, resulting in decreased polymer molecule chain mobility.

### 3.2. SEM Observation

**Figure 6:**
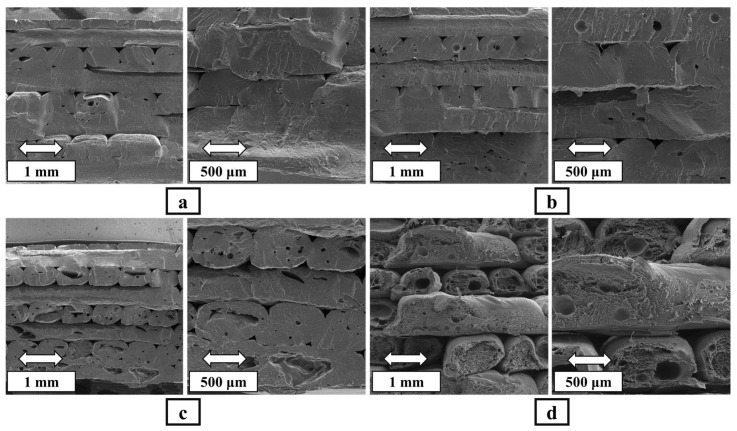
SEM images of the fracture surfaces of the printed specimens: (**a**) 0% Fe_3_O_4_, (**b**) 10% Fe_3_O_4_, (**c**) 15% Fe_3_O_4_, and (**d**) 20% Fe_3_O_4_.

Figure 6 displays cross-sectional images of 3D-printed PETG–ABS blend and PETG–ABS–Fe_3_O_4_ nanocomposites. According to the SEM images, the holes can be divided into three categories: holes in the junction of grids, in the junction of layers and inside the grids. According to Figure 6, in all samples, the interface between the grids is well established and the intertwining of the grids is evident for both the PETG–ABS blend (Figure 6a) and the PETG–ABS nanocomposite containing 10% iron oxide (Figure 6b). Although the connection quality of the grids decreases with increased nanoparticles, for the nanocomposite containing 20% iron oxide, these holes are more visible between the grids.

There are three main reasons for the formation of all three categories of holes: the nature of FDM printing, the rheological properties of the material, and the printing parameters. Briefly, microholes result from factors such as incomplete fluidity, fluctuation, rapid contraction of the grids, low printing temperature, and high melt strength, which are common challenges in FDM. The presence of these microholes compromises the interface quality between the layers, the printing ability, and the strength of the samples by creating local strains and porosity [32]. The printing of semisolid thin rasters on the previous layer is associated with shrinkage, which causes the cross-sectional area to shrink, resulting in weakly connected rasters. Selecting appropriate printing parameters can solve this problem to some extent by reducing the shrinkage rate through increasing the melt flow and increasing the bed temperature. In this article, we utilized a direct granule-based FDM printer equipped with a pneumatic system to control the melt flow to address the challenges associated with using a high weight percentage of nanoparticles and lack of control over geometry for producing precise and continuous filament. This helped to achieve a high-quality grid connection.

The second group consists of holes located between the layers, which appear in a triangular shape based on the raster angle. These microholes are present in nearly all samples at consistent ratios and densities for nanocomposites [32]. The origin of their formation is the circular cross section of the nozzle and the rheological properties of the material. Triangular microholes are formed at the top and bottom of the junction of grids with a circular cross section. A smoother flow of liquid material over the previous layer and higher temperatures can help somewhat reduce these holes [33,34]. The third category pertains to microholes in the grids, which are intensified by the presence of nanoparticles and cause porosity and pores within the grid itself. These holes have increased with the increase in the number of nanoparticles. In nanocomposites containing 15% and 20% iron oxide nanoparticles, these micropores play an essential role in the loss of mechanical properties. The images reveal a layered structure with microholes in all three nanocomposites. However, the sample containing 10% Fe_3_O_4_ nanoparticles by weight exhibits better print quality due to fewer microholes. This improvement can be attributed to the more uniform distribution of Fe_3_O_4_ nanoparticles in the polymer matrix.

**Figure 7:**
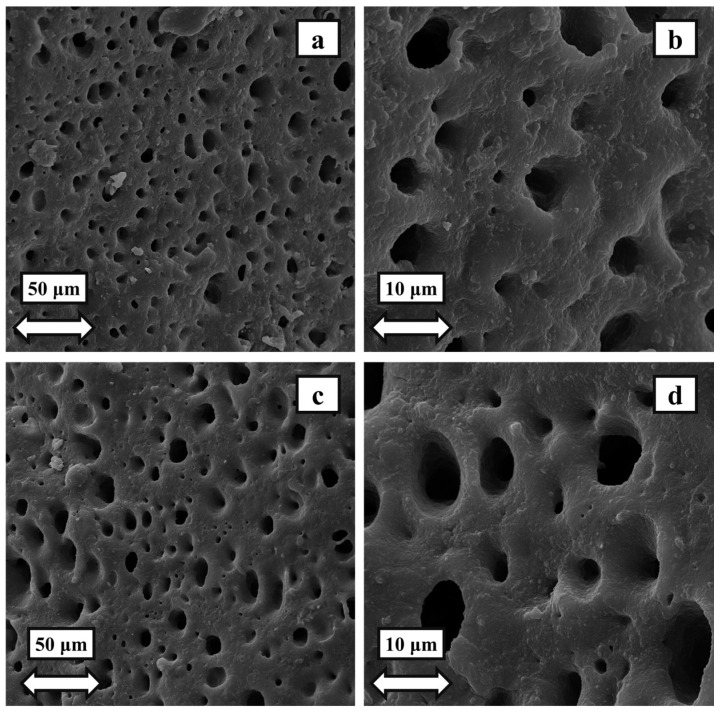
SEM image showing the morphology of: (**a**,**b**) PETG–ABS blend and (**c**,**d**) PETG–ABS–Fe_3_O_4_20.

In Figure 7, the morphology of the PETG–ABS blend is compared to that of the PETG–ABS–Fe_3_O_4_ nanocomposite. Based on the SEM images, the morphology of PETG-ABS appears as matrix droplets. In the morphology of two-phase blends, the secondary phase can exist in three distinct states. The first state is known as matrix-droplet morphology, in which the secondary phase is present in droplet form. The second stage is called co-continuous droplet morphology, in which the secondary phase is present in the form of continuous droplets located in a single path. The third state is referred to as the sea-island morphology, in which the secondary phase is present in the form of continuous droplets that are oriented in different directions. According to Figure 7, the addition of iron oxide nanoparticles does not affect the morphology of the matrix. Also, the addition of Fe_3_O_4_ does not affect the base polymer’s compatibility, a finding corroborated by DMTA analysis.

**Figure 8:**
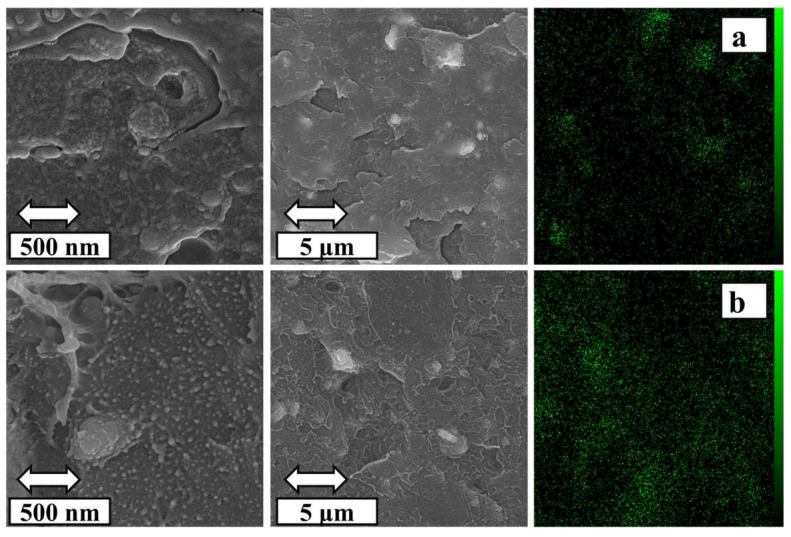
SEM image showing the morphology and EDX map of PETG–ABS–Fe_3_O_4_ nanocomposites with varying weight percentages of Fe_3_O_4_: (**a**) 10% Fe_3_O_4_, (**b**) 20% Fe_3_O_4_.

The distribution of iron oxide nanoparticles in PETG–ABS matrix with varying Fe_3_O_4_ contents is illustrated in Figure 8. The results of EDX analysis show that the distribution of iron oxide nanoparticles in the composite containing 10% is acceptable compared to other nanocomposites, and an almost uniform distribution of nanoparticles is observed. With the increase in iron oxide to 20% by weight, this balance is disturbed, and in most areas, clumping and a high volume of nanoparticles are observed. The results of this quantitative study indicate that composites containing 20% iron oxide display a higher aggregation of iron oxide compared to the 10% sample. It is true that this evaluation covers a small segment of the morphology, but these results are confirmed by SEM and mechanical properties. These results can be confirmed by the SEM images with lower magnification. In fact, according to Figure 6, the amount of porosity inside the grids for the composite containing 20% increases strongly, which is due to the areas of lumping. Another reason that supports the findings of EDX is the decrease in mechanical properties as the iron oxide content increases.

### 3.3. Mechanical Properties

**Figure 9:**
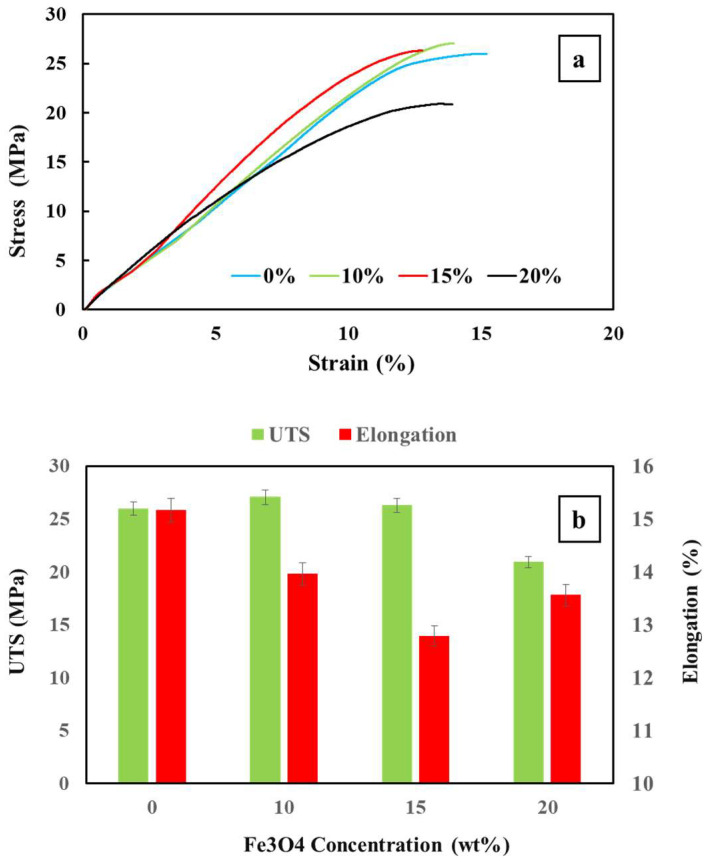
The behavior of PETG–ABS–Fe_3_O_4_ nanocomposites in terms of Fe_3_O_4_ concentration under tensile mode: (**a**) stress–strain curves and (**b**) calculated strength and elongation.

An investigation was conducted to evaluate the impact of Fe_3_O_4_ on the mechanical properties of composites printed with FDM in the tensile loading mode. The study compared the results of the stress–strain diagram for the pure PETG–ABS blend and PETG–ABS–Fe_3_O_4_ nanocomposite samples reinforced with 10%, 15%, and 20% by weight of Fe_3_O_4_. These results are depicted in Figure 9 and Table 2. The findings revealed that adding Fe_3_O_4_ nanoparticles up to 15% by weight increased the tensile stress while reducing elongation. Specifically, the tensile stress increases from 25.98 MPa for the pure PETG–ABS sample to 26.3 MPa and 27.05 MPa for the 10% and 15% Fe_3_O_4_ nanocomposites, respectively. This increase in tensile strength is accompanied by a decrease in elongation from 15.15% to 13.94% and 12.78%. In the sample reinforced with 10% by weight of nanoparticles, the modulus is nearly equal to that of a pure PETG–ABS sample, resulting in a completely brittle fracture for this nanocomposite. However, the elongation has decreased compared to the sample without nanoparticles, indicating that the nanocomposites have become more brittle due to the addition of Fe_3_O_4_. In the nanocomposite with a weight percentage of 20%, there is a decrease in tensile strength compared to the nanocomposite with a weight percentage of 10% and pure PETG–ABS blend samples. This decrease can be attributed to the higher weight percentage of nanoparticles. The reason for this is that as the weight percentage of Fe_3_O_4_ increases in the polymer matrix, the tendency of these nanoparticles to accumulate and form lumps in the composition also increases. SEM results also confirmed the uneven distribution of nanoparticles in this sample compared to the sample with 10% by weight. Increasing the amount of Fe_3_O_4_ carries a greater risk of creating areas with uneven distribution, which in turn are susceptible to stress concentration. This susceptibility can weaken the mechanical properties and reduce the tensile strength. Hung [20] analyzed the impact of Fe_3_O_4_ on the mechanical properties of PLA–ENR–Fe_3_O_4_ polymer blends, and these findings support our results. Similarly, when it comes to PETG–ABS blends and iron oxide-reinforced nanocomposites, nanocomposites with 20% by weight of Fe_3_O_4_ exhibit the lowest strength. As the Fe_3_O_4_ content increases, both the tensile strength and elongation decrease. The decrease in tensile strength is more noticeable in the sample containing 20% Fe_3_O_4_ than the 10% sample. However, the ultimate tensile strength of the nanocomposites ranges from 20 MPa to 27 MPa, indicating that a suitable and acceptable distribution of nanoparticles up to 15% by weight is achieved in the polymer matrix. In addition to the distribution of nanoparticles, the printability of the matrix and the adhesion of the interface between the blend content and the nanoparticles are also of great importance [1,10,22]. As mentioned, an increase in nanoparticles reduces the possibility of uniform distribution, and the same issue has a strong impact on printing ability [10]. The increase in nanoparticles and their clumping causes clogging of the nozzle and reduction in the melt flow rate during printing, and in this way, it is effective in reducing the mechanical properties. Therefore, the increase in nanoparticles up to 15% by weight has a reinforcing role and causes a continuous increase in tensile strength. However, for the PETG–ABS–Fe_3_O_4_ nanocomposite containing 20% iron oxide, due to the clumping of iron oxide nanoparticles and the subsequent reduction in printing quality, the mechanical properties drop drastically and reach a value lower than the pure PETG–ABS blend.

### 3.4. Shape-Memory Effect

**Figure 10:**
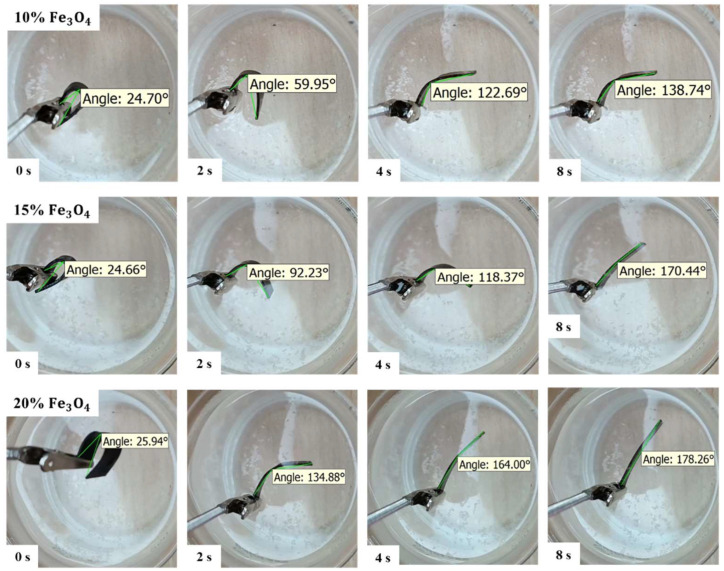
Recovery steps according to time with direct thermal stimuli for PETG–ABS–Fe_3_O_4_ nanocomposites with varying weight percentages of Fe_3_O_4_.

The shape-memory cycle involved examining shape fixity and shape recovery under two modes of direct and indirect thermal stimulation. Figure 10 shows the stage of direct shape recovery over time in boiling water for all three PETG–ABS–Fe_3_O_4_ nanocomposites. The analysis of shape-memory properties of PETG–ABS–Fe_3_O_4_ nanocomposites reveals that all samples exhibit a 100% shape fixity ratio. PETG exhibits good shape fixity, and the addition of ABS with a higher transition temperature and elastic modulus helps the net points of PETG [28,35]. Another noteworthy point is the quick recovery of nanocomposites. As already mentioned, amorphous SMPs such as PETG have a weak netpoint, mainly due to molecular entanglements, and strengthening them can improve shape-memory performance for industrial applications. According to Figure 10, the shape recovery process is accomplished in less than eight seconds for different PETG–ABS–Fe_3_O_4_ nanocomposites.

**Figure 11:**
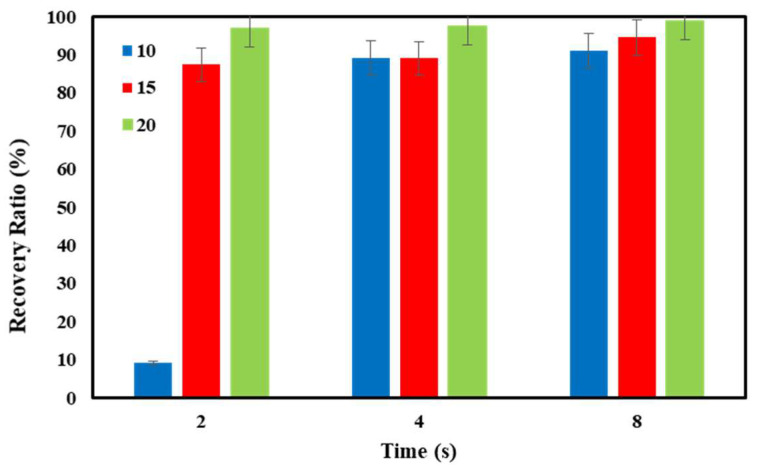
Results of percentage shape recovery over time for PETG–ABS–Fe_3_O_4_ nanocomposites through direct stimulation.

In Figure 11, the results of shape recovery ratios over time at the recovery temperature of 100 °C are presented for PETG–ABS–Fe_3_O_4_ nanocomposites. According to the results, the shape recovery rate increases significantly with the increase in the weight percentage of Fe_3_O_4_. The shape recovery ratios for PETG–ABS–Fe_3_O_4_10, PETG–ABS–Fe_3_O_4_15, and PETG–ABS–Fe_3_O_4_20 nanocomposites are 91.50%, 94.55% and 98.95%, respectively. The presence of iron oxide nanoparticles not only increases heat transfer but also leads to faster shape recovery due to the higher elastic moduli they provide. This result is consistent with previous research, showing that the rate of improvement is directly related to the increase in iron oxide nanoparticles. These findings are also consistent with previous research.

**Figure 12:**
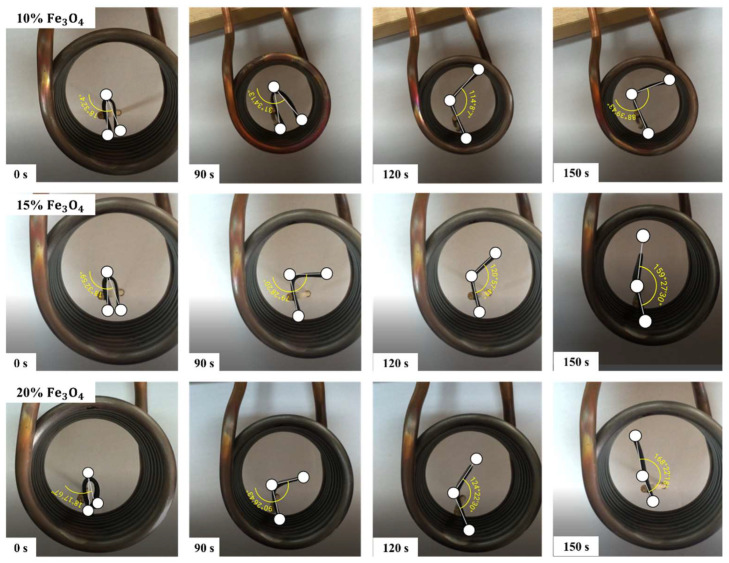
Recovery steps of PETG–ABS–Fe_3_O_4_ nanocomposites through indirect stimulation.

**Figure 13:**
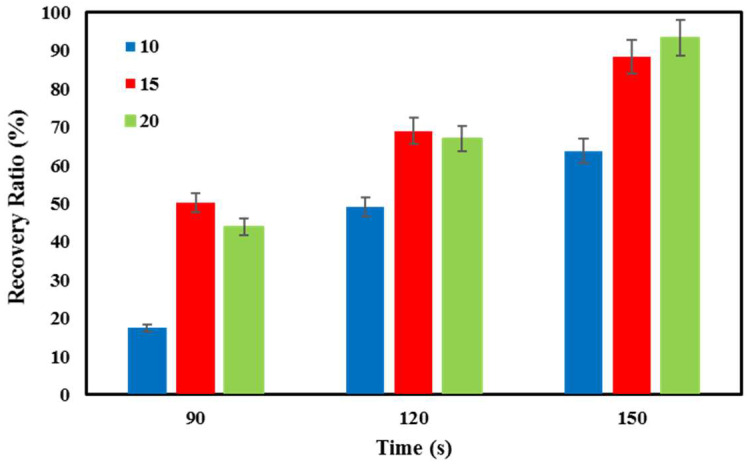
Results of percentage shape recovery over time for PETG–ABS–Fe_3_O_4_ nanocomposites through indirect stimulation.

Figure 12 shows the shape recovery steps of PETG–ABS–Fe_3_O_4_ nanocomposites through indirect stimulation. Figure 13 provides quantitative data on indirect shape recovery, showing that the amount of recovery increases with time for each nanocomposite. The results reveal a clear correlation between the degree of shape recovery and the concentration of Fe_3_O_4_. Specifically, nanocomposites containing 10%, 15%, and 20% by weight exhibit impressive shape recovery of 63.77%, 88.48%, and 93.33%, respectively. Moreover, the results demonstrate a significant increase in the shape recovery rate with higher weight percentages of Fe_3_O_4_. The increase in magnetic nanoparticles increases the eddy current, and this increases the heating rate in the material containing these nanoparticles [16].

## 4. Conclusions

For the first time, a PETG–ABS–Fe_3_O_4_ nanocomposite was manufactured with a matrix consisting of 70% PETG and 30% ABS, along with varying percentages (10%, 15%, and 20%) of iron oxide nanoparticles during the melt mixing process. This innovative material was then processed and 3D/4D-printed. The study involved analyzing shape-memory properties, mechanical properties, thermal properties, and microstructure of the nanocomposites through shape recovery under direct and indirect stimulation, uniaxial tensile tests, dynamic thermomechanical analysis (DMTA), and scanning electron microscopy (SEM). To overcome the challenges related to filament production and printing of nanocomposites with a high filler weight percentage, we utilized a granule-based FDM printer equipped with a pneumatic system. This approach allowed us to regulate the melt flow and provide acceptable printability. The results of the SEM investigation proved that the print quality decreased with the increase in iron oxide. The increase in iron oxide was associated with a decrease in the quality of the connections between grids and layers. Most importantly, it caused high porosity and pores inside the grids for nanocomposites containing 15% and 20% by weight of iron oxide. The results of the EDX analysis showed that the distribution of iron oxide nanoparticles in the composite containing 10% was acceptable, with an almost uniform distribution of nanoparticles observed. With an increase in iron oxide to 20% by weight, the balance was disrupted, resulting in clumping and a high volume of nanoparticles in most areas. The ultimate tensile strength (UTS) increased from 25.98 MPa for the pure PETG–ABS sample to 26.3 MPa and 27.05 MPa for the 10% and 15% Fe_3_O_4_ nanocomposites, respectively. The increase in tensile strength was accompanied by a decrease in elongation from 15.15% to 13.94% and 12.78%. In the PETG–ABS–Fe_3_O_4_ nanocomposite containing 20% iron oxide, the clumping of iron oxide nanoparticles led to a significant reduction in printing quality, causing the mechanical properties to drop drastically below those of the pure PETG–ABS blend. The results of direct and indirect shape recovery studies show that increasing the amount of iron oxide nanoparticles improves the SME. It reduces the recovery time and increases the recovery ratio. Of course, this effect is more intense in magnetic field excitation, as the eddy current is strengthened with the increase of ferromagnetic particles.

## Figures and Tables

**Table 1:** Variable parameters in 3D printing by FDM of PETG–ABS–Fe_3_O_4_ nanocomposites.

Printing Parameters	Values
Velocity (mm/min)	250
Nozzle Temperature (°C)	230 ± 10
Bed Temperature (°C)	50
Nozzle Diameter (mm)	0.6
Layer Thickness (mm)	0.45
Printing Direction	0–90
Air Pressure (Bar)	2.5–4
Infill Density (%)	100

**Table 2:** Quantitative results extracted from the tensile test.

Samples	Tensile	
	UTS (MPa)	El (%)
10% Fe_3_O_4_	27.05	13.9
15% Fe_3_O_4_	26.30	12.8
20% Fe_3_O_4_	20.94	13.5

## Data Availability

Data are included within the article.
